# The Clinical Features and Diagnosis of Canavan’s Disease: A Case Series of Iranian Patients

**Published:** 2014

**Authors:** Parvaneh KARIMZADEH, Narjes JAFARI, Habibe NEJAD BIGLARI, Elham RAHIMIAN, Farzad AHMADABADI, Hamid NEMATI, Mohamad Mehdi NASEHI, Mohammad GHOFRANI, Mohsen MOLLAMOHAMMADI

**Affiliations:** 1 Pediatric Neurology Research Center, Shahid Beheshti University of Medical Sciences, Tehran, Iran; 2 Pediatrics, Pediatric Neurology Research Center, Shahid Beheshti University of Medical Sciences, Tehran, Iran; 3 Neuroradiologist, Haghighat Radiology Center, Tehran, Iran; 4 Pediatric Neurologist, Ardabil University of Medical Sciences, rdebil, Iran; 5 Pediatric Neurology Department, Hazrat Fatemeh Masoumeh Hospital, Qom University of Medical Sciences, Qom, Iran

**Keywords:** Canavan’s disease, diagnosis. N- Acetylaspartic acid, Magnetic Resonance Spectrometry

## Abstract

**Objective:**

Canavan’s disease is a lethal illness caused by a single gene mutation that is inherited as an autosomal recessive pattern. It has many different clinical features especially in the non-Ashkenazi Jewish population.

**Material & Methods:**

45 patients were referred to the Pediatric Neurology Department of Mofid Children’s Hospital in Tehran-Iran from 2010–2014 with a chief complaint of neuro developmental delays, seizures, and neuroimaging findings of leukodystrophy were included in this study. Magnetic Resonance Spectrometry (MRS) and neuro metabolic assessment from a referral laboratory in Germany confirmed that 17 patients had Canavan’s disease.

**Results:**

Visual impairment, seizure, hypotonia, neuro developmental arrest, and macrocephaly were the most consistent findings in the patients in this study. Assessments of neuro developmental status revealed that 13 (76%) patients had neuro developmental delays and 4 (24%) patients had normal neuro development until 18 months of age and then their neuro developmental milestones regressed. In this study, 100% of cases had macrocephalia and 76% of these patients had visual impairment. A history of seizures was positive in 8 (47%) patients and began around 3 months of age with the most common type of seizure was tonic spasm. EEGs were abnormal in all epileptic patients. In ten of the infantile group, we did not detect elevated level of N-acetylaspartic acid (NAA) in serum and urine. However, the MRS showed typical findings for Canavan’s disease (peaks of N-acetylaspartic acid).

**Conclusion:**

We suggest using MRS to detect N-acetylaspartic acid as an acceptable method for the diagnosis of Canavan’s disease in infants even with normal serum and urine N-acetylaspartic acid levels.

## Introduction

Canavan’s disease is a fatal illness that begins early in infancy and the major manifestation is white matter dysmyelination that are delineated as spongy degenerations. Initially, Canavan’s described was described by Canavan in 1931 and is now known as Canavan’s disease. The striking features of Canavan’s disease is a failure to attain neuro developmental milestones, abnormally decreased muscular tone, increased head circumference, seizure, atrophy of the optic nerve, and early death (1-6).

Canavan’s disease inherited with an autosomal recessive pattern and is most common in the Ashkenazi Jewish community but there are other populations with different ethnic backgrounds who get this disease. Canavan’s disease is caused by a mutation in the gene that encodes the aspartoacylase enzyme. This enzymes main role is converting N-acetylaspartic acid to aspartate and acetate in the brain. 

Cloning the gene that is responsible for aspartoacylase enzyme production has lead to finding mutations that cause Canavan’s disease. There are many known mutations and some of them are more common to specific ethnic groups. 

When converting the N-acetylaspartic acid is blocked because of aspartoacylase enzyme deficiency, then N-acetylaspartic acid accumulates in the white matter and complicates normal development (7-12).

The exact pathophysiology of Canavan’s disease is not elucidated but there are some interesting hypotheses that myelin breakdown can be the main cause of consequent devastating events. Management of the disease is based on supporting nutrition and controlling seizures and infections but gene therapy is a promising new horizon in treatment and many investigators try to transfer normal gene sequences in the patient’s genome. This study evaluates the clinical features of Canavan’s disease and elucidates the best approach for a correct diagnosis (13-20).

## Materials & Methods

**Participants and diagnosis**

In this study, 45 patients (aged from 5–66 months) referred to the Pediatric Neurology Department of Mofid Children’s Hospital in Tehran-Iran (2010–2014) with a chief complaint of neuro developmental delay, seizures, and neuroimaging findings of leukodystrophy were enrolled in our study. 

The exclusion criteria was patients who had leukodystrophy in their brain Magnetic resonance Imaging (MRI), but Tandem mass spectrometry in serum, gas chromatography in urine showed increased metabolites except N-acetylaspartic acid, and the MRS detected material sediment of other than NAA or borderline results.

Canavan’s disease was confirmed in 17 patients from clinical manifestation, neuroimaging findings, elevated level of NAA in MRS, and neuro metabolic assessments from a referral laboratory in Germany.

**Patient information**

Patient information such as age, gender, past medical history, developmental status, general appearance, clinical, and neuroimaging findings were collected. 

**Management**

Management included control of seizures, treatment for infection (if present), and follow up of growth index by the best feeding drive.

**Data analysis**

The data was analyzed using descriptive methods and no statistical testing was applied. This is an observational study.

**Ethical approval**

Institutional ethical approval to conduct this study was obtained from the Pediatric Neurology Research Center of Shahid Beheshti University of Medical Sciences, Tehran, Iran. All parents signed a written consent for participation in the study.

## Results

**Patient information**

Canavan’s disease was confirmed in 17 patients (10 males and 7 females).

The earliest case was diagnosed in a 5-month-old patient and the latest case was diagnosed in a 5.5-year-old patient. The median age of presentation of symptoms was 8 months and the median age of patients at assessment time was 31 months. A total of 85% of patients (14 patients) were offspring of consanguineous marriages. Nine patients had parents who were first cousins and in another 5 patients, their parents were second cousins. A family history of Canavan’s disease was positive for 2 patients.

**Clinical results**

A prenatal history of patients showed there was a history of preeclampsia in one patient and history of gestational diabetes in another patient. Prenatal history revealed that one patient was preterm with a gestational age of 32 weeks, one patient was post term with gestational age of 41 weeks, and others were term with normal weight and head circumference at birth. At neonatal period, 6 (35%) patients had jaundice that was treated with phototherapy. The patient who was delivered prematurely because of preeclampsia was admitted into the neonatal intensive care unit for 11 days. Congenital anomalies were not detected in any newborn babies at birth. Assessments of patients neuro developmental status, revealed 13 (76%) patients had neuro developmental arrest and 6 (24%) had normal neuro developmental milestones until 18 months of age and then they showed regression.

A history of seizure was positive in 8 (47%) patients and began around 3 months of age and the most common type of seizure was a tonic spasm. Three patients had drug resistant epilepsy. A neurological examination showed that 2 patients had ataxia, 2 had tremors, and 3 had severe dystonia. 11 (64%) patients had spasticity with increased deep tendon reflex. Increased deep tendon reflex and spasticity was more commonly observed in patients who were more than 1 year old. On the other hand, the deep tendons reflex was normal in 6 (36%) infant patients that all of them showed hypotonia. Ophthalmic examination revealed visual impairment in 41% of patients. Between 7 (41%) patients had impaired fix and follow, 6 (35%) patients were blind with optic atrophy from the Fundoscopic examination. Not all patients had a history of macrocephalia but all of patients had macrocephalia at 5 months old (above 95% percentile).

Based on the growth index assessment, eleven patient had failure to thrive (between 3–10 percentile). A followup for all patients showed intellectual disabilities. One patient had a sibling with Canavan’s disease as well. There was no certain point in other physical examinations, such as hair and skin, face, chest, and abdomen.

**Para clinical results**

From the lab data, one patient had transitory increased levels of AST and ALT; and metabolic acidosis was not seen in patients. Electroencephalography (EEG) in 8 (47%) patients with seizures was abnormal with no special patterns. The EMG-NCV study was normal in all patients.

A potential assessment of abnormal vision in the majority of patients was present. Serum amino acid levels examined by the tandem mass spectrometry method, venues blood gas analysis and levels of lactate, ammonia, and pyruvate were within normal limits. N-acetylaspartic acid (NAA) levels in the serum of one patient was abnormally high but its concentration in urine of 6 (36%) patients was high (the levels of NAA in cases were in a range of 410–1050 μmol/mol creatine; with control level of 41 μmol/mol creatine). A genetic study was done on two brothers who were 5 months and 4 years old and revealed a positive result for c.244–245 in the SA mutation was found.

**Neuroimaging results**

**Fig 1:**
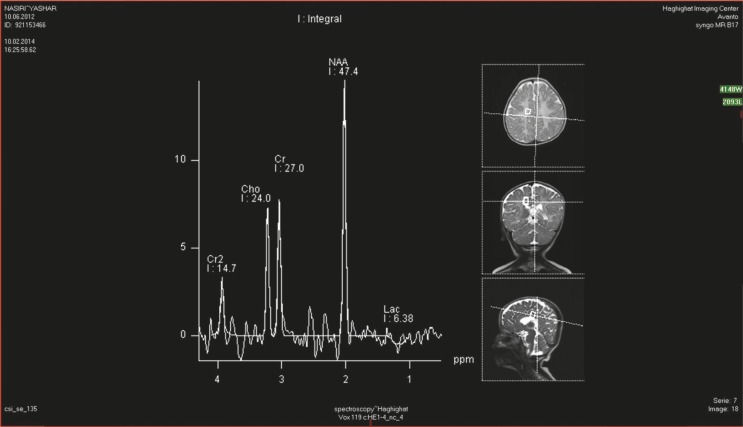
Detection of the markedly elevated NAA peak on MR spectroscopic images in patients with Canavan’s disease as well as an increase in the NAA-to-choline ratio and the NAA-to-creatine ratio. These are considered indicative of Canavan’s disease given associated white matter abnormalities

In all patients, the MRS revealed a high peak of N-acetylaspartic acid but normal urine N- acetylaspartic acid level was seen in 9 patients under one years of age (Fig 1).

**Fig 2:**
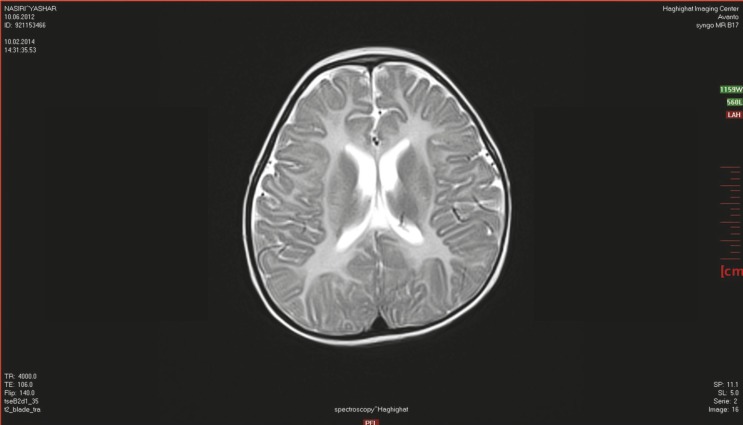
Canavan’s disease demonstrates bilateral symmetric T2 white matter hyperintensity including involvement of the subcortical arcuate fibers. This disease appears diffusely throughout the cerebral white matter and does not enhance in computed tomography (CT) or MR imaging, and demonstrates variable involvement of the basal ganglia and cerebellar white matter

This finding can be explained by the accumulation of N-acetylaspartic acid in the brain occurs before increasing its concentration in the urine. MRI in all patients showed subcortical-periventricular white matter leukodystrophy. 

However, in 3 (18%) patients, there was bilateral globus pallidus and thalamus signal changes. (Fig 2).

## Discussion

Michals and Matalon indicated that 117studied patients concluded the mean concentration of N-acetylaspartic acid in urine of patients with severe Canavan’s disease was 1440.5 ± 873.3 μmol/mmol creatine. 

They highlighted the elevation of N-acetylaspartic acid concentration in CSF and in the urine of patients but the concentration of NAA were similar to our study (21). 

Breitbach-Faller et al. indicated that brain ultrasonography of patients with Canavan’s disease and presence of white matter echogenicity (22).

Matalon et al in two separate studies about neuroimaging of Canavan’s disease concluded that CTs or MRIs in infancy period may interpreted as normal (23), but in older patients’ the prominent findings are symmetric diffuse cortical and subcortical white matter changes although cerebellar and brain stem involvement may be less commonly seen (24). These studies about neuroimaging findings also support our findings. To the best of our knowledge, there are limited studies (with small sample size) about using MRS in the literature. Our study has a significant number of patients identified from the use MRS of Canavan’s disease.

In conclusion, one of the interesting findings in our study was that psychomotor arrest in the first 6 months of age and primary hypotonia replaced by spasticity with age. Both of these findings can be clinical manifestations of Canavan’s disease and our study showed that MRI and MRS are valuable diagnostic evaluations of this disease. An MRI can show dysmyelination of subcortical–periventricular white matter and the MRS shows N-acetylaspartic acid accumulation in the brain that can be detected before increasing its concentration in the urine, which is suitable for infants when N-acetylaspartic acid cannot be found in the urine.

We suggest the use of MRS for early diagnosis of Canavan’s disease in infants when N-acetyl Aspartic acid in the early stages of disease cannot be found in the urine. 

The MRS can show the peak of N-acetyl Aspartic Acid in hyper signal region of the white matter. **Competing interests: **None declared.

**Funding: **The authors received no financial support for the research and publication of this article.
